# Supplementary material for: Turing structuring with multiple nanotwins to engineer efficient and stable catalysts for hydrogen evolution reaction
Source: Nat Commun. 2023 Sep 4;14:5389. doi: 10.1038/s41467-023-40972-w (PMC10477283; doi:10.1038/s41467-023-40972-w)
Supplement: Supplementary file 1 — Supplementary Information [file 41467_2023_40972_MOESM1_ESM.pdf]

Supplementary Information

Turing structuring with multiple nanotwins to engineer efficient and stable catalysts for hydrogen evolution reaction

AUTHORS: Jialun Gu[1, 2, 6, 7]†, Lanxi Li[1, 3, 7]†, Youneng Xie[1, 2, 7], Bo Chen[4], Fubo Tian[5], Yanju Wang[1, 2, 7], Jing Zhong[3], Junda Shen[3, 7], Jian Lu[1, 2, 3, 6, 7]*

[1]Centre for Advanced Structural Materials, City University of Hong Kong Shenzhen Research Institute, Greater Bay Joint Division, Shenyang National Laboratory for Materials Science, Shenzhen, China.

[2]Department of Mechanical Engineering, City University of Hong Kong, Tat Chee Avenue, Hong Kong, China.

[3]Department of Materials Science and Engineering, City University of Hong Kong, Tat Chee Avenue, Hong Kong, China.

[4]Department of Chemistry, City University of Hong Kong, Tat Chee Avenue, Hong Kong, China.

[5]State Key Laboratory of Superhard Materials, College of Physics, Jilin University, Changchun 130012, People's Republic of China

[6]CityU-Shenzhen Futian Research Institute, No. 3, Binglang Road, Futian District, Shenzhen, China.

[7]Hong Kong Branch of National Precious Metals Material Engineering Research Centre, City University of Hong Kong, Hong Kong, China.

†These authors contributed equally to this work.

*Corresponding author. Email: jianlu@cityu.edu.hk

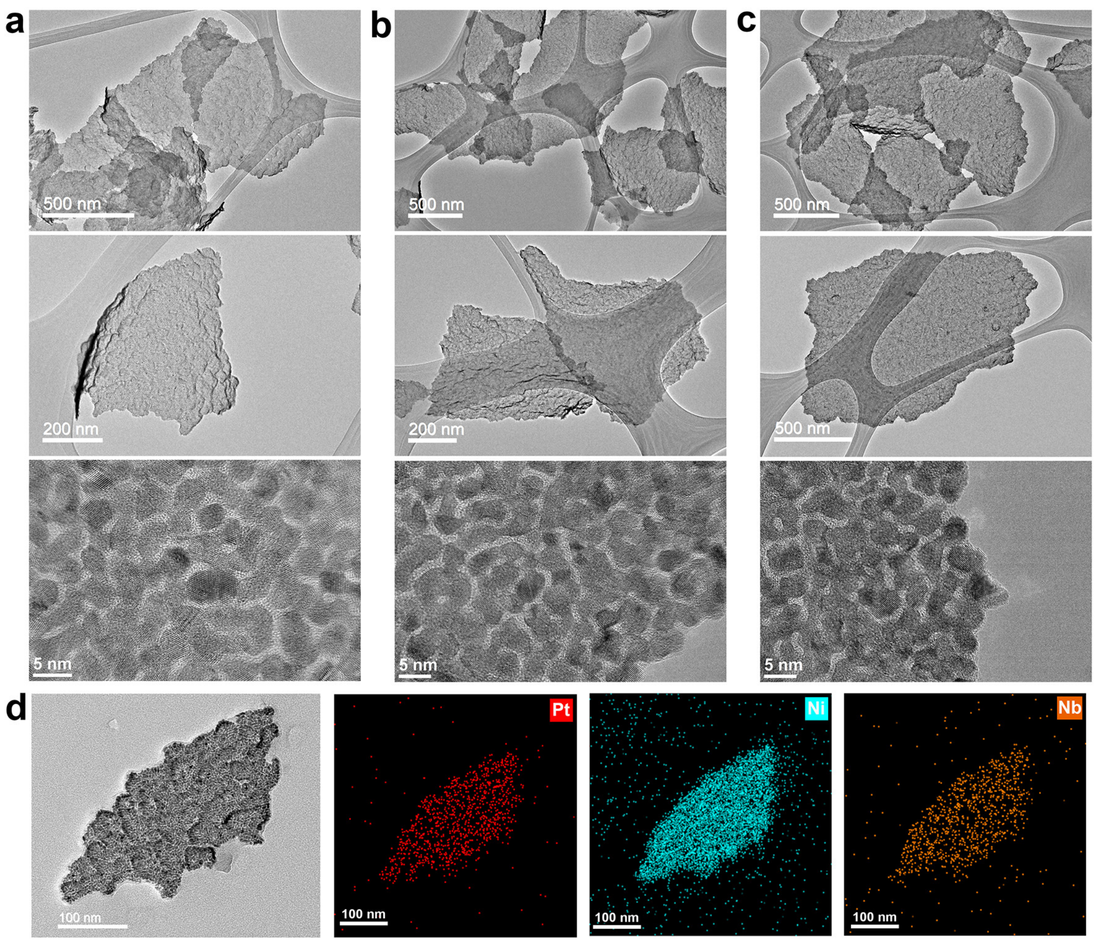

Supplementary Fig. 1 | The morphology, structure and composition of Turing PtNiNb. TEM images and HRTEM images of the free-standing Turing PtNiNb nanosheets, the thickness of Turing PtNiNb nanosheets are **a**, 4 nm, **b**, 8 nm and **c**, 12 nm. The three PtNiNb samples exhibit similar characterization of Turing structure. **d**, Elemental mapping of platinum, nickel and niobium in a single Turing PtNiNb nanosheet.

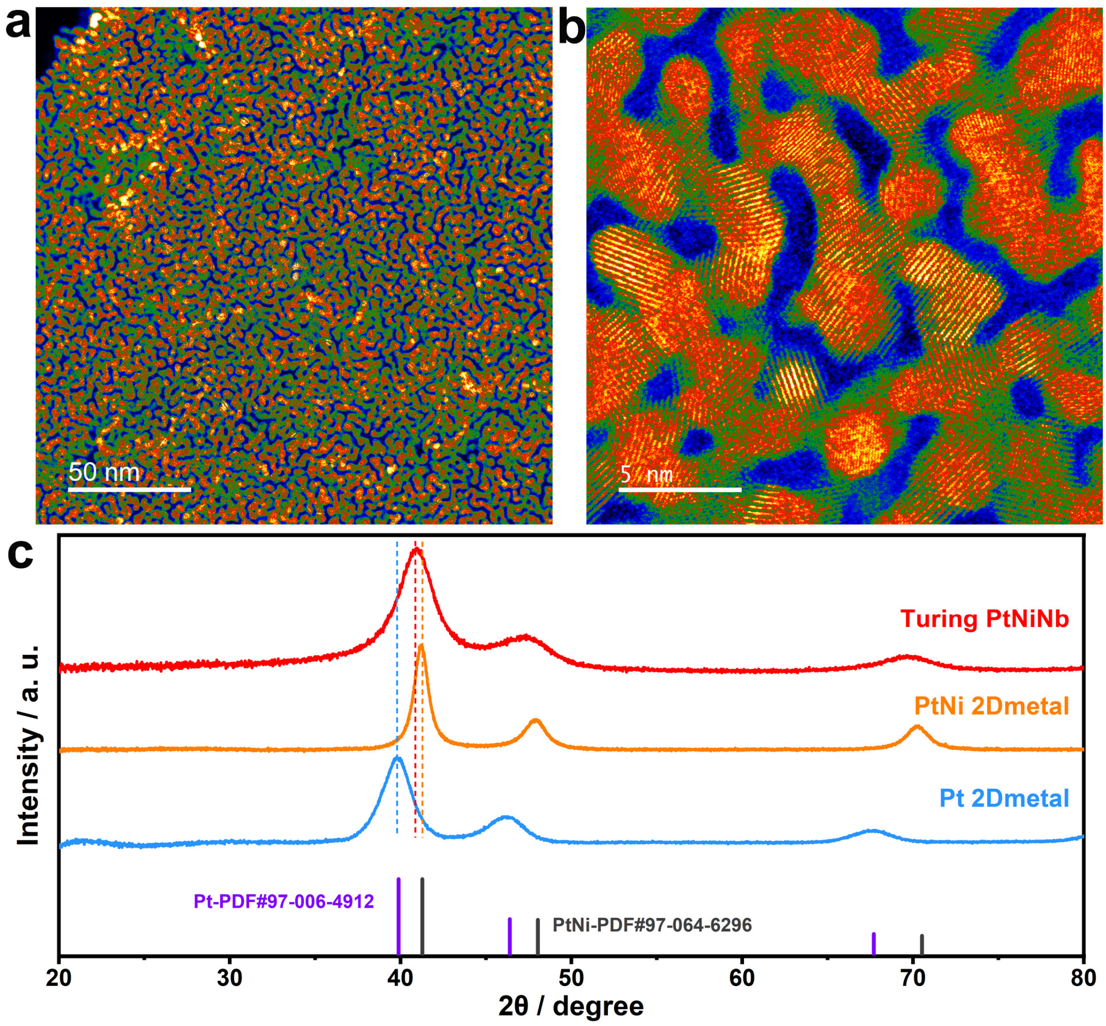

Supplementary Fig. 2 | a, **b**, The HAADF-STEM images with different magnifications of Turing PtNiNb nanosheet, showing Turing-type patterns. **c**, XRD spectra of the Turing PtNiNb, PtNi 2D metal and nanosheets and monometallic Pt 2D metals. The peak positions of Pt and PtNi from standard database are marked for comparison.

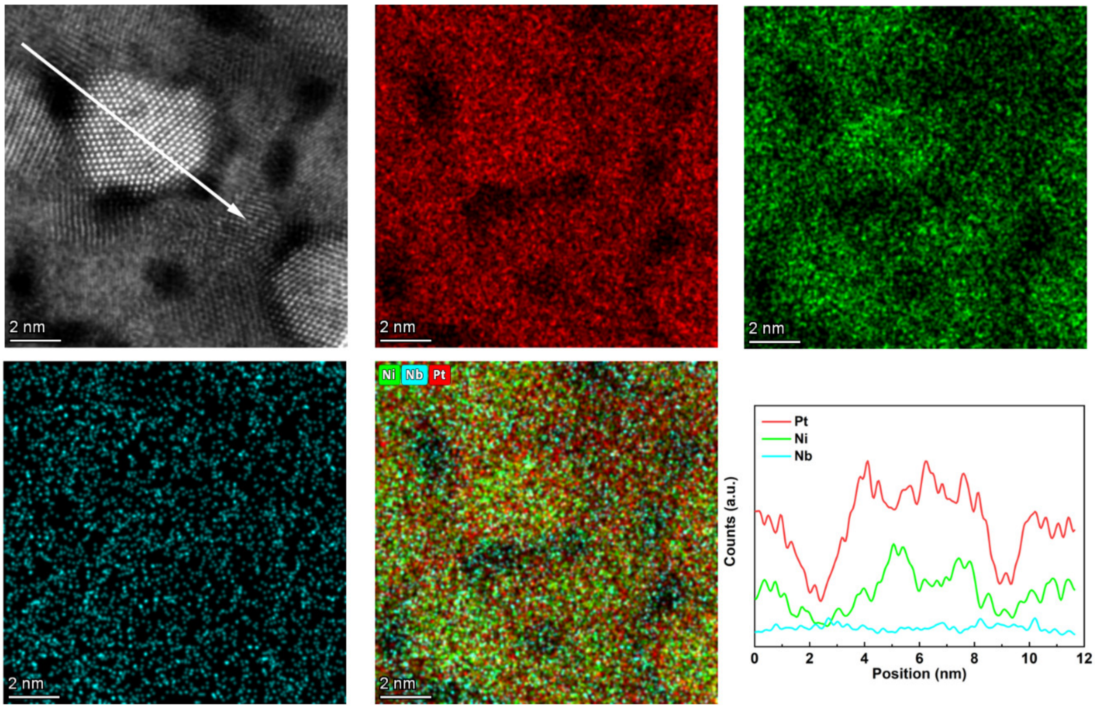

Supplementary Fig. 3 | Elemental mapping and line-scanning of platinum, nickel and niobium in a Turing PtNiNb nanosheet, the white arrow represents the line-scanning direction.

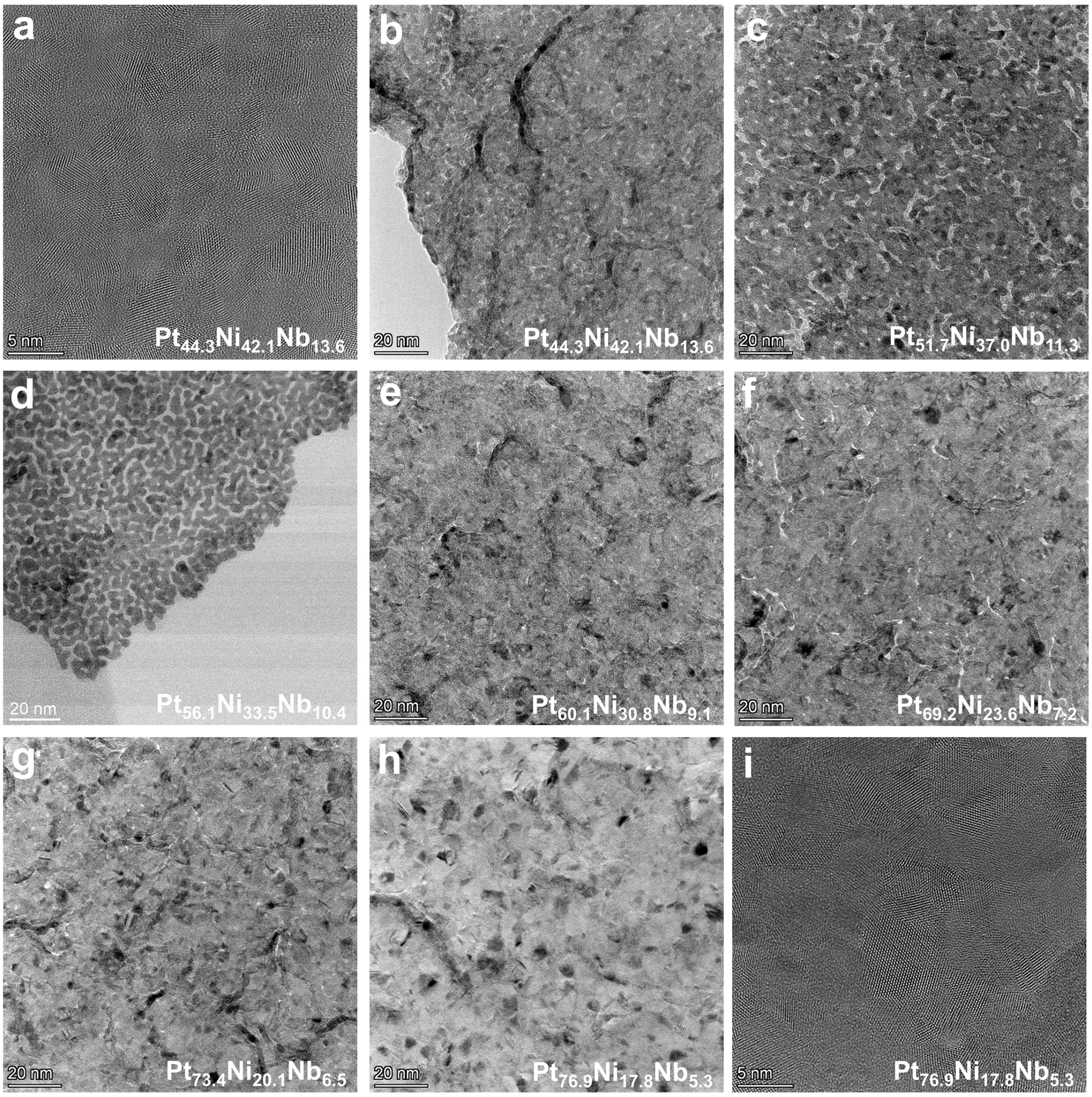

Supplementary Fig. 4 | a, The HRTEM image of $Pt_{44.3}Ni_{42.1}Nb_{13.6}$ nanosheet. TEM images of $Pt_{44.3}Ni_{42.1}Nb_{13.6}$ nanosheet (b), $Pt_{51.7}Ni_{37.0}Nb_{11.3}$ nanosheet (c), Turing PtNiNb (d), $Pt_{60.1}Ni_{30.8}Nb_{9.1}$ nanosheet (e), $Pt_{69.2}Ni_{23.6}Nb_{7.2}$ nanosheet (f), $Pt_{73.4}Ni_{20.1}Nb_{6.5}$ nanosheet (g), $Pt_{76.9}Ni_{17.8}Nb_{5.3}$ nanosheet (h). **i**, The HRTEM image of $Pt_{76.9}Ni_{17.8}Nb_{5.3}$ nanosheet.

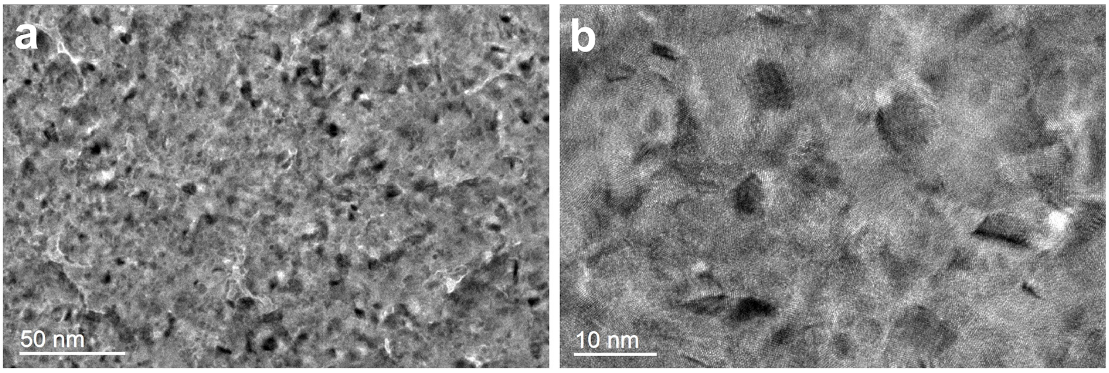

Supplementary Fig. 5 | a, The TEM image of bimetal PtNi nanosheet. **b**, The HRTEM image of bimetal PtNi nanosheet.

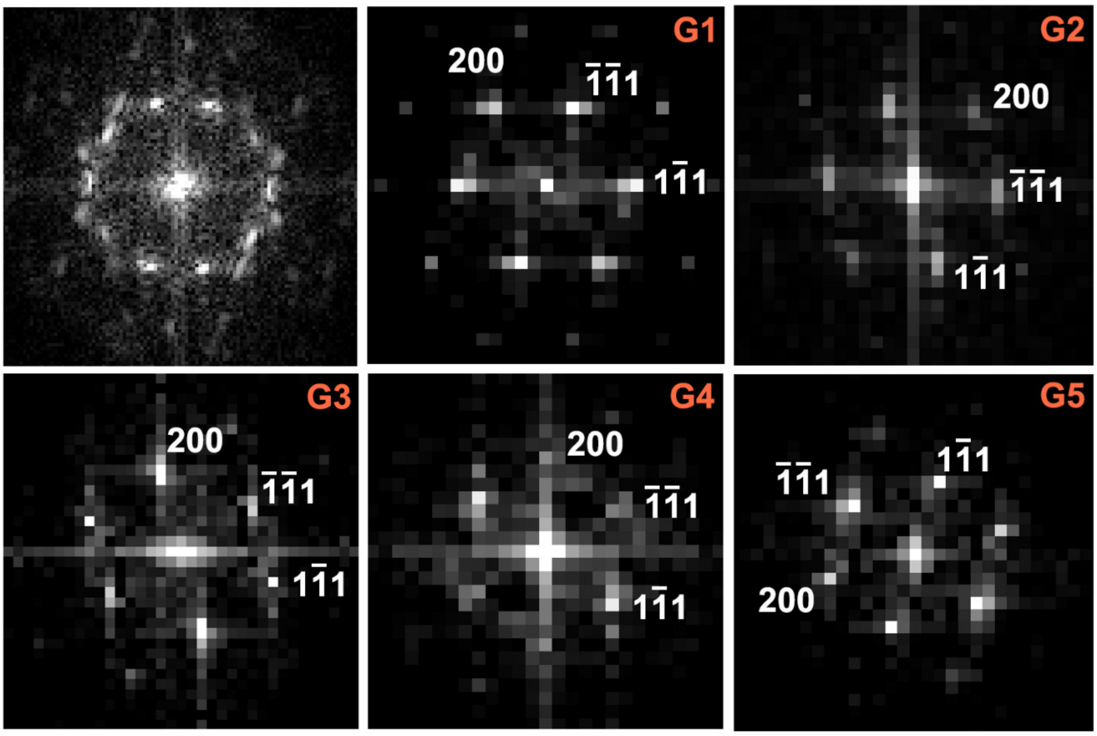

Supplementary Fig. 6 | Fast Fourier transform patterns of grains (G1, G2, G3, G4 and G5) in the five-fold twins shown in Fig. 2a.

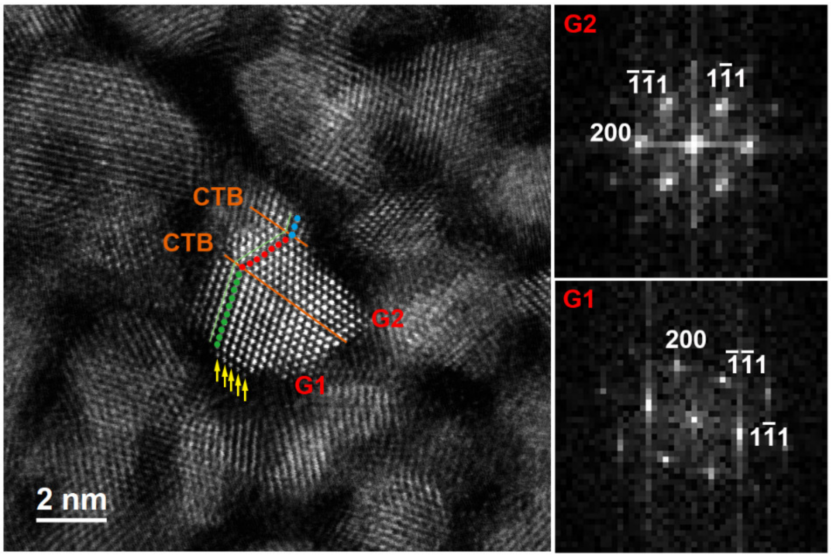

Supplementary Fig. 7 | The HAADF-STEM image of a twin-junction with parallel coherent twin boundaries. The orange line represents the coherent boundary. The FFT images of twins G1 and G2 are shown in the right panel.

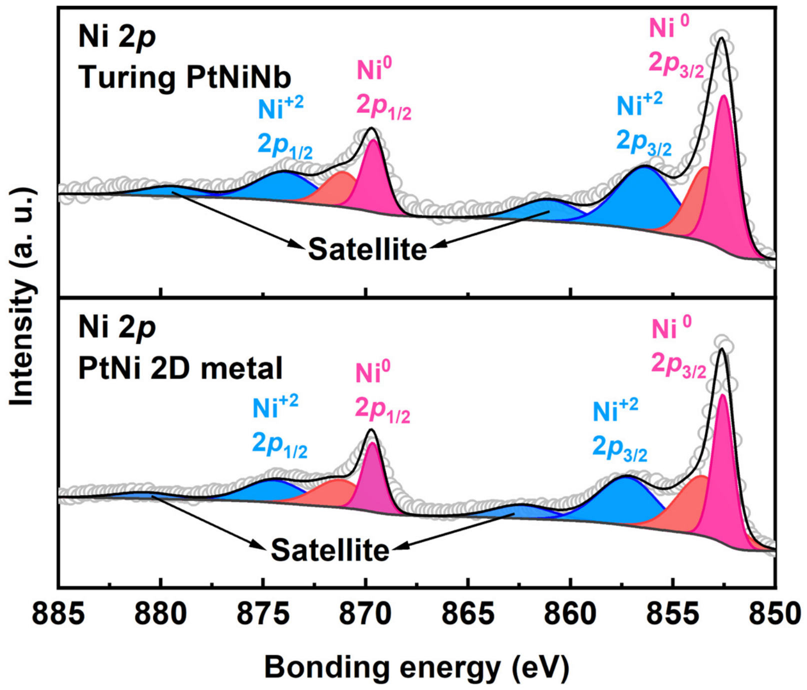

Supplementary Fig. 8 | High resolution XPS spectra comparison for Ni 2*p* in Turing PtNiNb (the up panel) and PtNi 2D metal (the bottom panel).

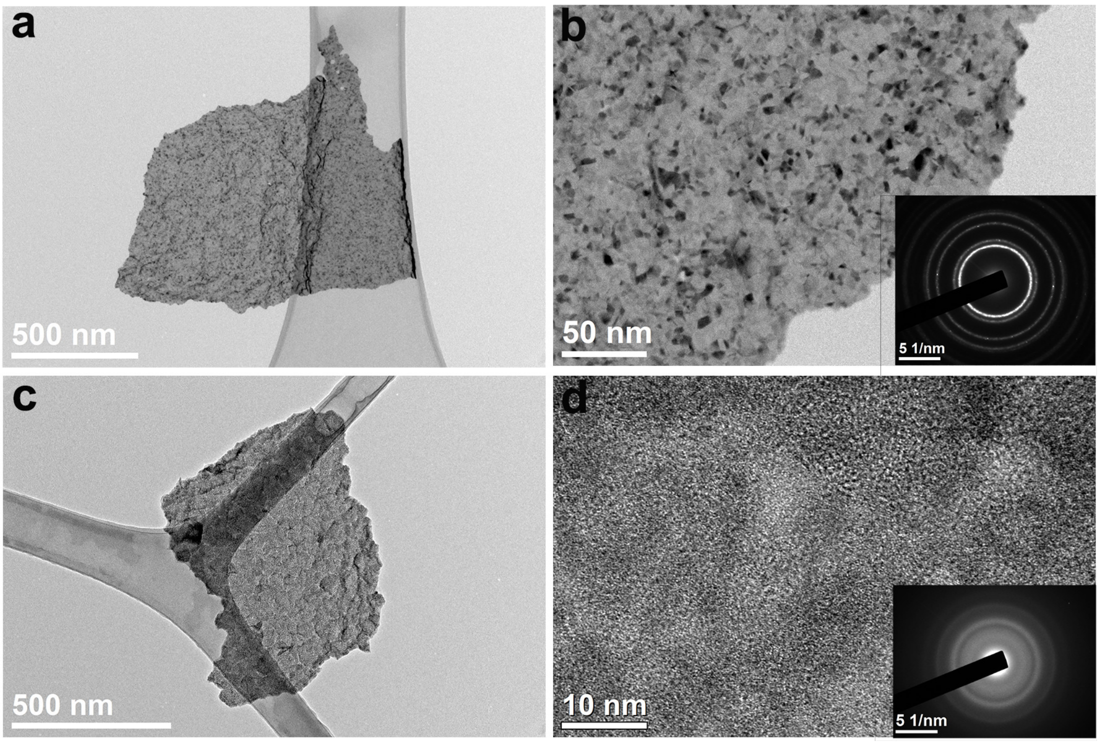

Supplementary Fig. 9 | a, **b**, TEM images of monometallic Pt 2D metal. Inset is the corresponding SAED patterns showing the sharp diffraction rings. The TEM results suggest that the monometallic Pt 2D metal is composed of nano-crystals with the grain-size smaller than 10 nm. **c**, TEM image of the NiNb 2D metal. **d**, HRTEM image of the NiNb 2D metal. Inset shows the corresponding SAED patterns. The maze-like patterns of atomic arrangement and the diffraction halos confirm the fully amorphous structure of the as-prepared NiNb 2D metal.

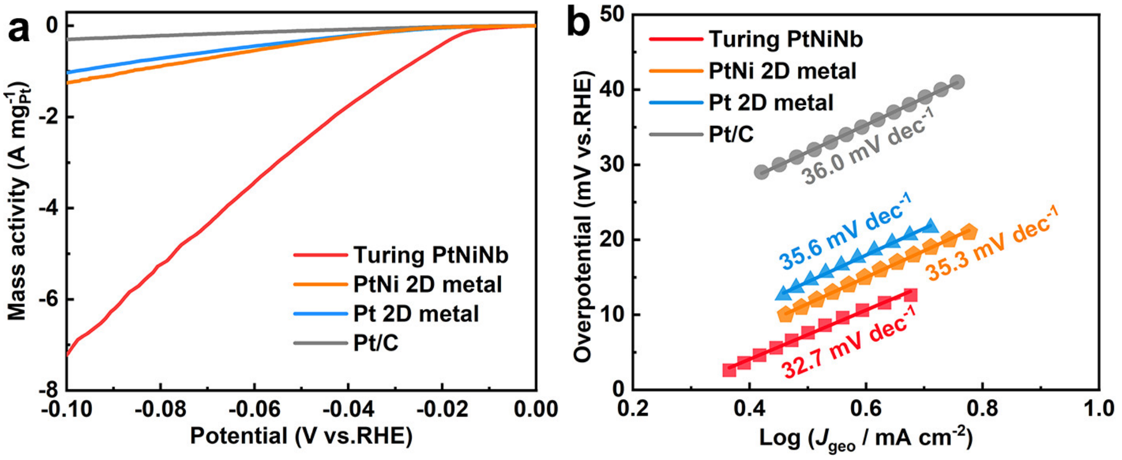

Supplementary Fig. 10 | a, LSV curves based on mass activity of Pt in Turing PtNiNb, PtNi 2D metal, Pt 2D metal and Pt/C. **b**, Tafel plots of Turing PtNiNb, PtNi 2D metal, Pt 2D metal and Pt/C.

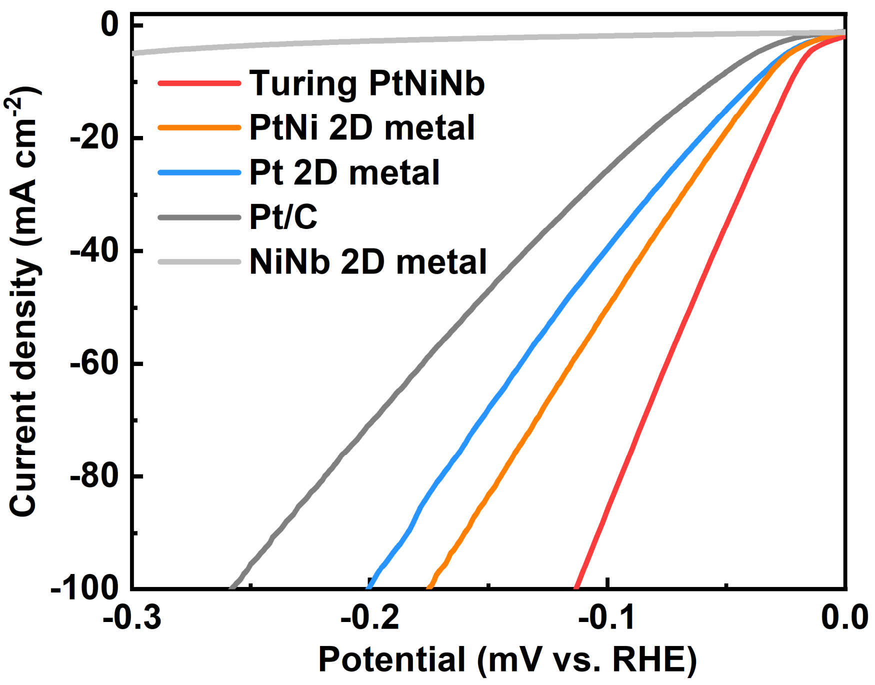

Supplementary Fig. 11 | LSV curves without iR compensation corresponding to curves in Fig. 4a.

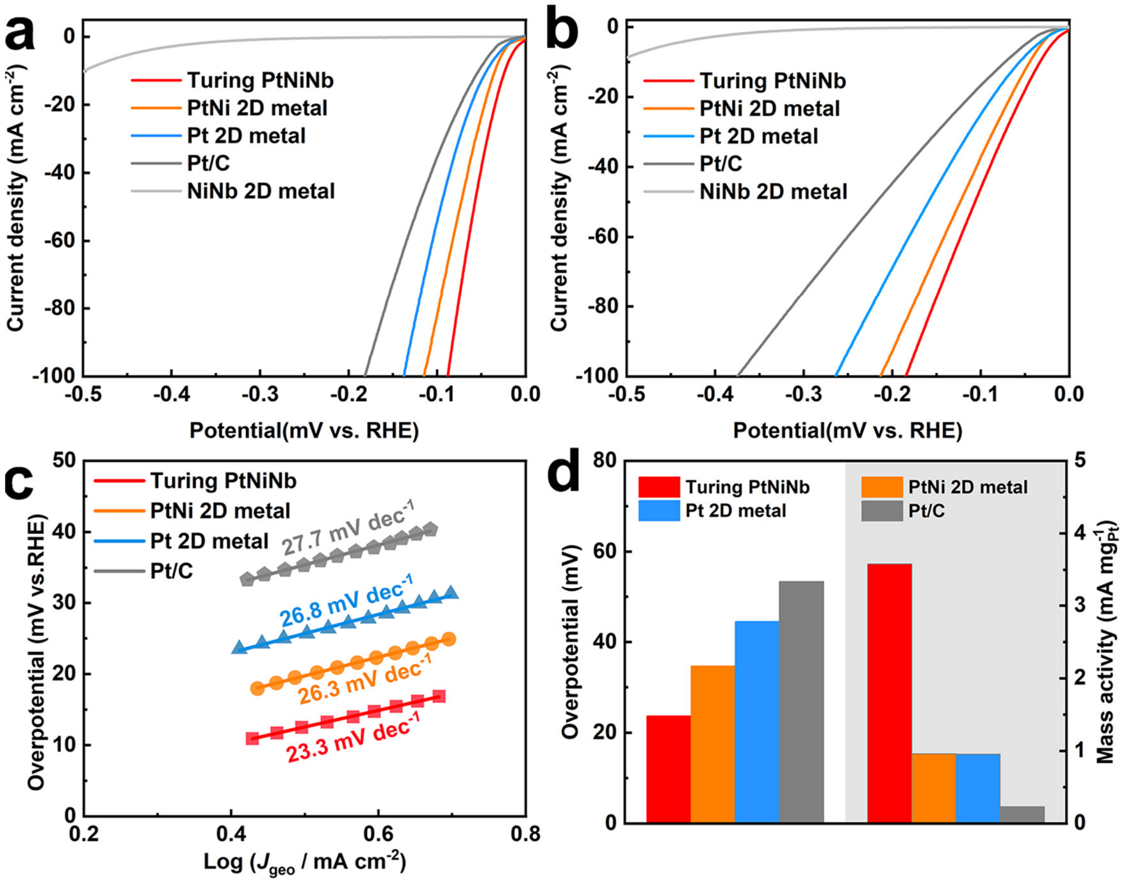

Supplementary Fig. 12 | Electrochemical performance tested on rotating disk electrode. a, LSV curves of Turing PtNiNb, Pt 2D metal, PtNi 2D metal, Pt/C and amorphous NiNb 2D metal in 1.0 M KOH, with 85% resistance compensation. **b**, LSV curves without resistance compensation. **c**, Tafel plots of Turing PtNiNb, Pt 2D metal, PtNi 2D metal and Pt/C. **d**, Comparison for overpotential at 10 mA cm^{-2} and mass activity at 100 mV (vs. RHE) between samples in (**a**).

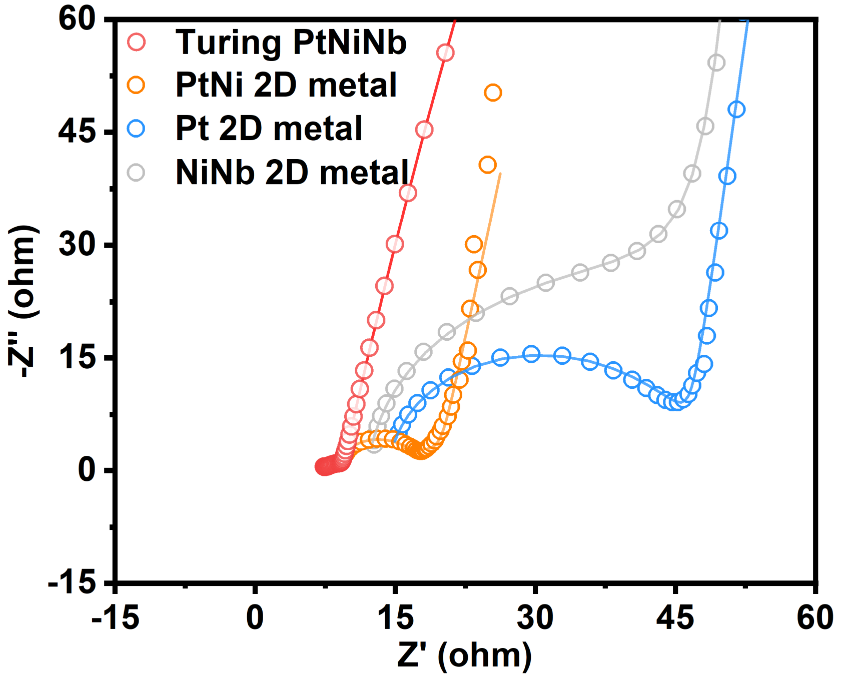

Supplementary Fig. 13 | Nyquist plots collected at an overpotential of 50 mV for Turing PtNiNb, PtNi 2D metal, Pt 2D metal and NiNb 2D metal.

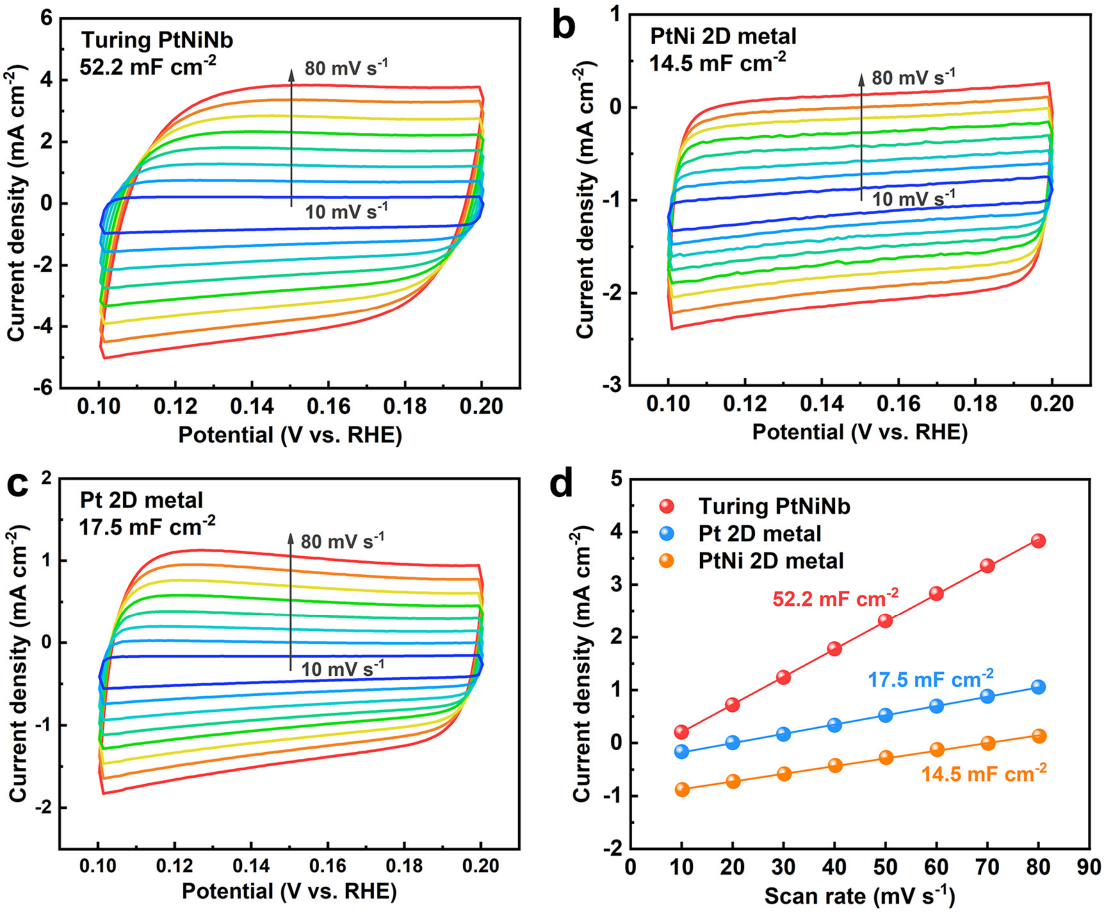

Supplementary Fig. 14 | CV curves measured at different scan rate from 10 to 80 mV s^{-1} in 1.0 M KOH for **a**, Turing PtNi **b**, PtNi 2D metal and **c**, Pt 2D metal. **d**, Capacitive current at middle potential of CV curves as function of scan rates correspondingly.

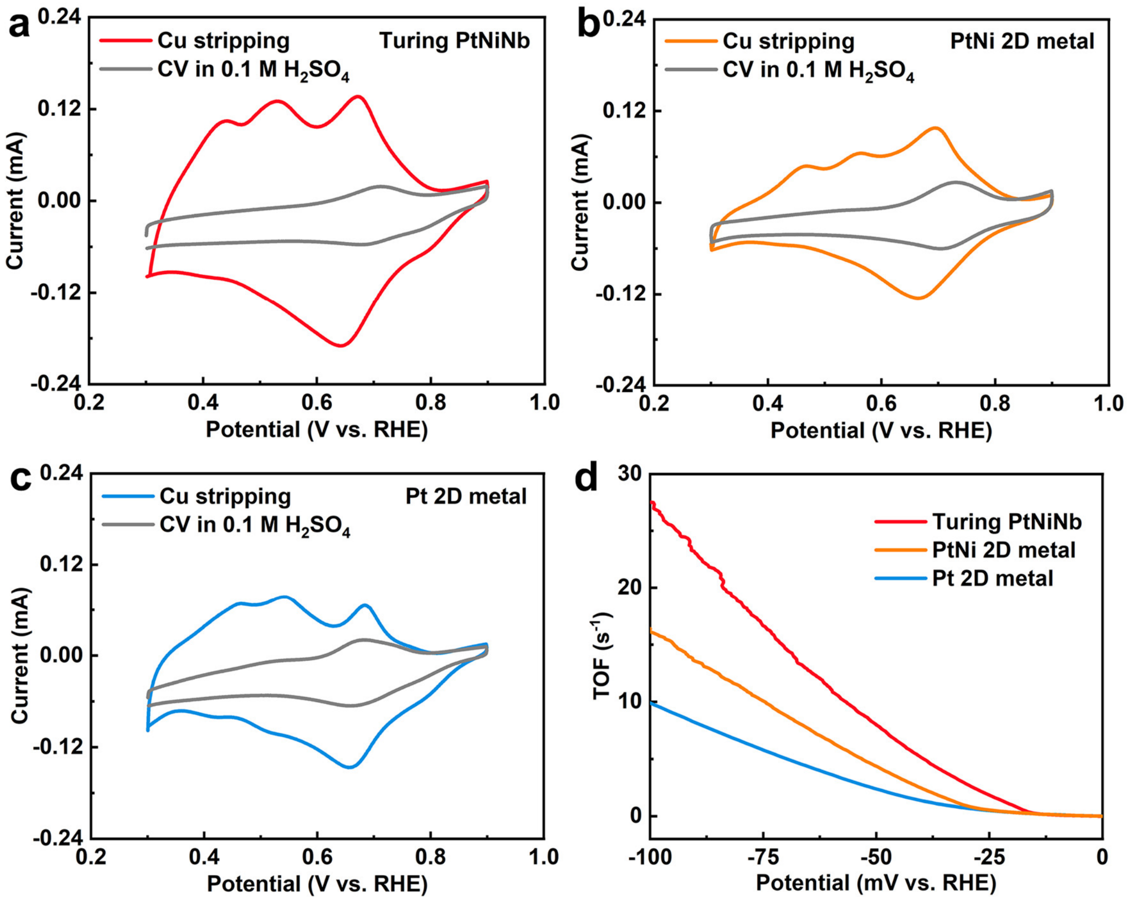

Supplementary Fig. 15 | CV curves recorded in 0.1 M H_2SO_4 before and after being polarized in 0.1 M H_2SO_4 + 5 mM $CuSO_4$ for **a**, Turing PtNiNb, b, PtNi 2D metal and **c**, Pt 2D metal. **d,** The potential-dependent TOF curves for Turing PtNiNb, PtNi 2D metal and Pt 2D metal.

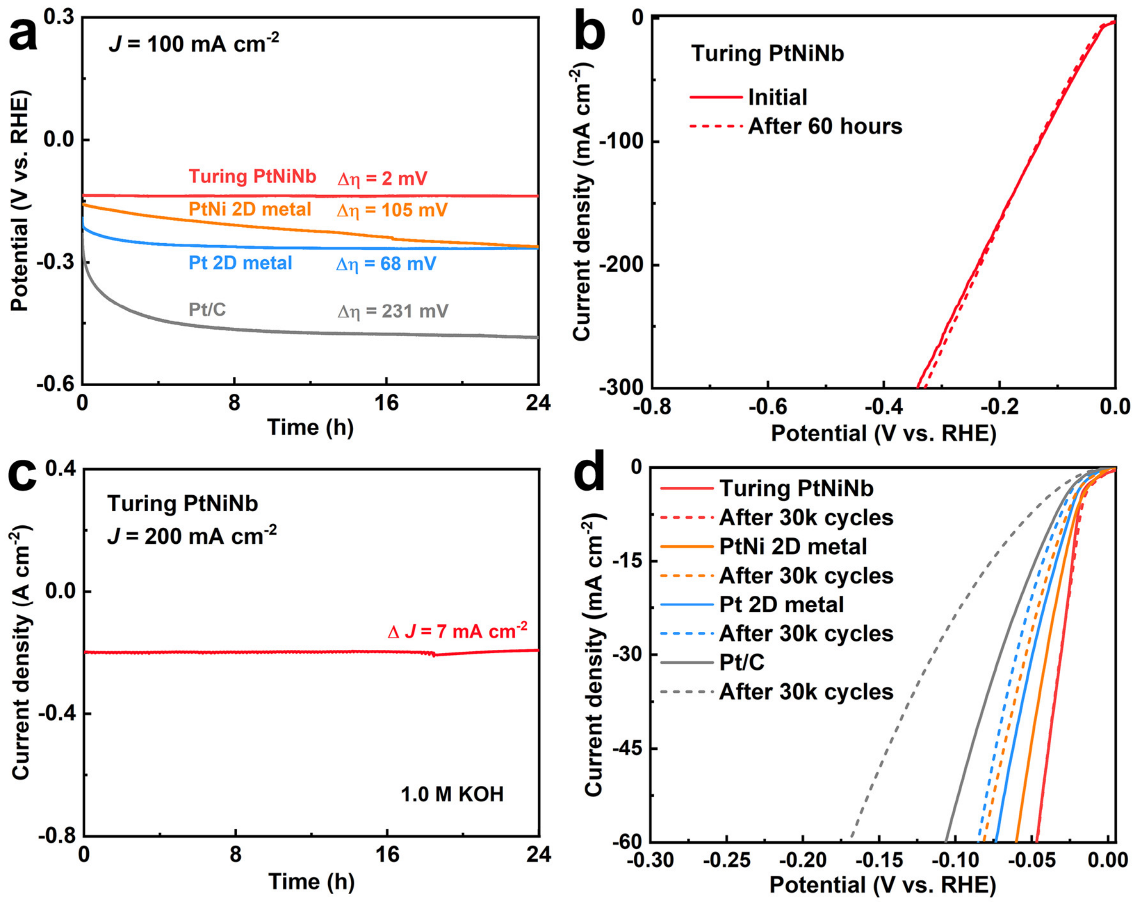

Supplementary Fig. 16 | a, Galvanostatic measurement in 1.0 M KOH continuing 24 hours at 100 mA cm^{-2} for Turing PtNi, PtNi 2D metal, Pt 2D metal and Pt/C. The galvanostatic curves are without iR compensation. **b**, The LSV curves comparison before and after 60-h galvanostatic measurement under 200 mA cm^{-2}. The LSV curves are without iR compensation. **c**, Potentiostatic measurement continuing 24 hurs at applied potential of -1.1221V for Turing PtNi. **d**, Comparison of LSV curves recorded before and after 30,000 cycles in ADT.

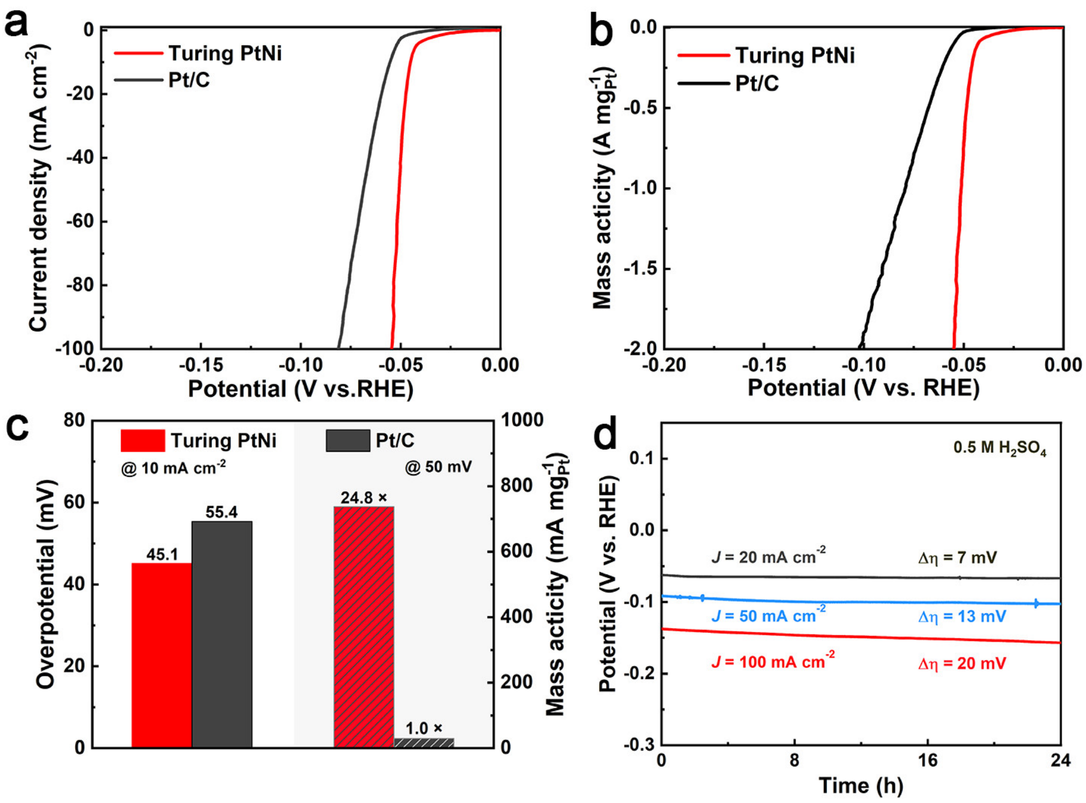

Supplementary Fig. 17 | a, LSV curves measured in 0.5 M H_2SO_4 of Turing PtNiNb and Pt/C. **b**, Mass activity measured in 0.5 M H_2SO_4 of Turing PtNiNb and Pt/C. **c**, Comparison for overpotential at 10 mA cm^{-2} and mass activity at 50 mV (vs. RHE) between Turing PtNiNb and Pt/C. **d**, Galvanostatic measurement in 0.5 M H_2SO_4 continuing 24 hours at 20, 50 and 100 mA cm^{-2} for Turing PtNiNb. The galvanostatic curves are without iR compensation.

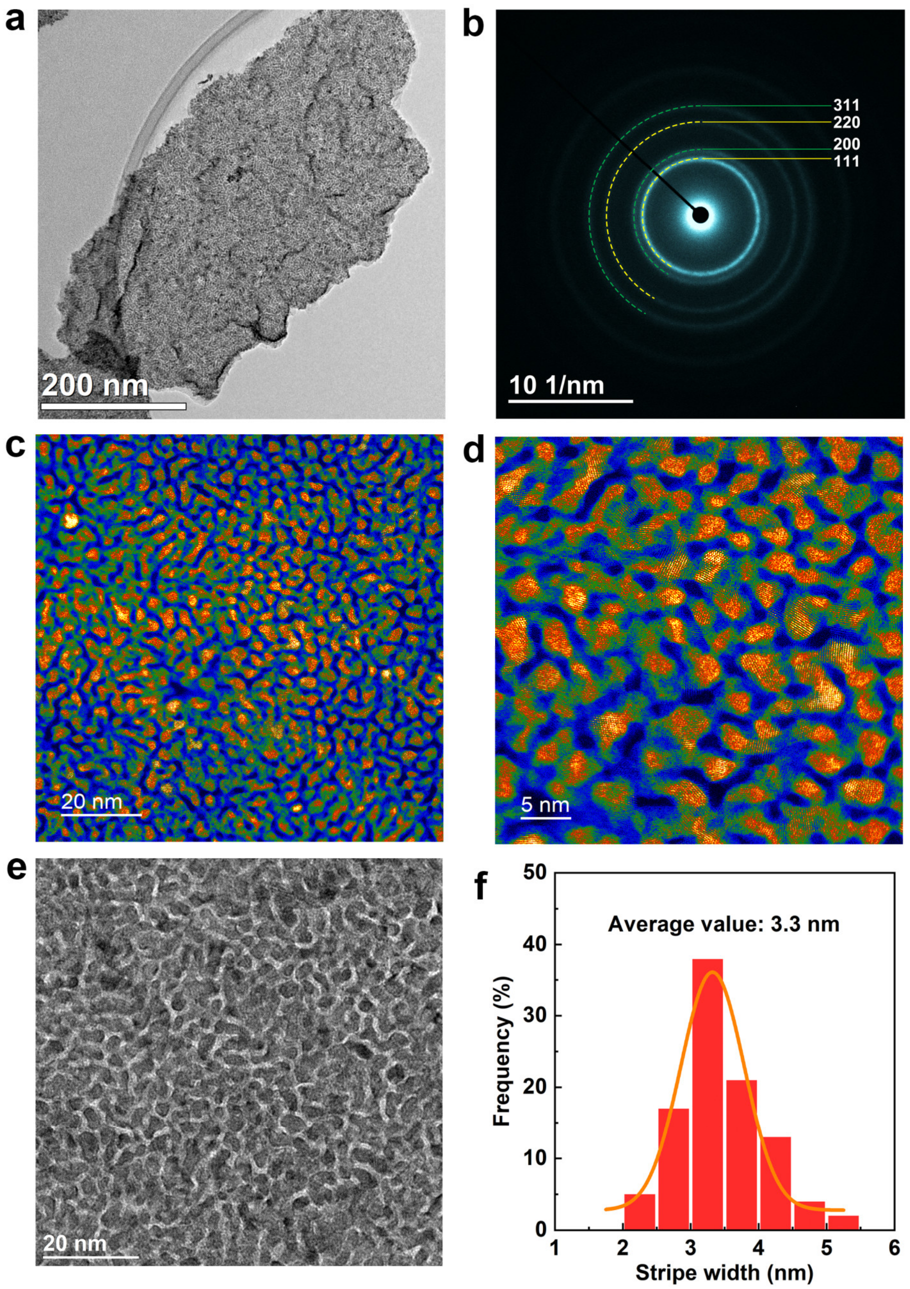

Supplementary Fig. 18 | The structure characterization of the Turing PtNiNb nanosheet after the long-time stability test (200 mA cm^{-2}, 60h) in 1.0 M KOH. a, Low-magnification TEM image showing a single slice of Turing PtNiNb after stability test. **b**, The SAED pattern of the tested Turing PtNiNb nanosheet, this result indicates the fcc structure as the same with the pristine Turing PtNiNb samples. **c**, **d**, The HAADF-STEM images. **e**, TEM images of the Turing PtNiNb nanosheet after stability test. The characterization of Turing patterns is clear presented in these TEM images. **f**, The size distribution of Turing stripes.

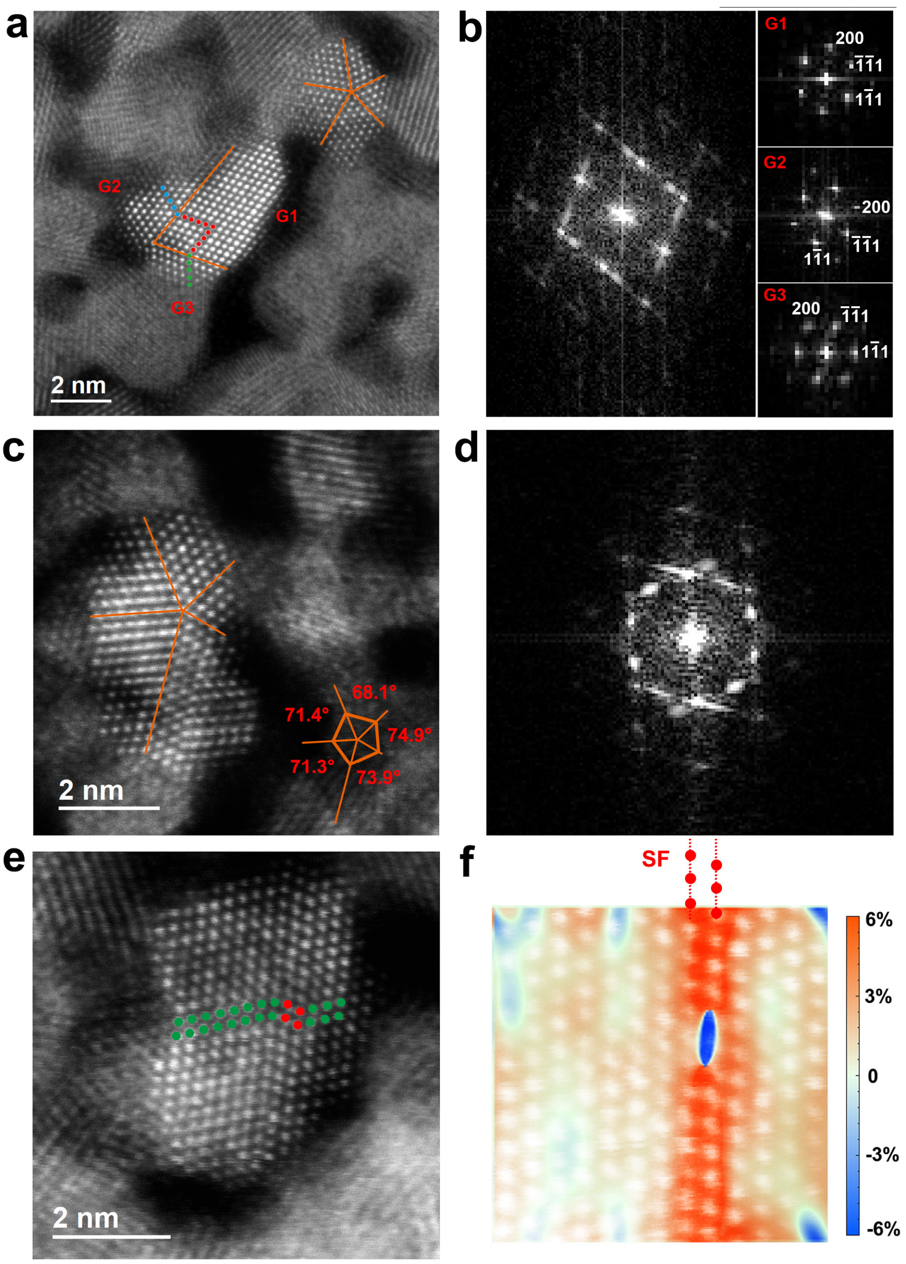

Supplementary Fig. 19 | Microstructure of Turing PtNiNb after the long-time stability test (200 mA cm^{-2}, 60h) in 1.0 M KOH. a, The HAADF-STEM image of a twin-junction with coherent twin boundaries and the fivefold twins. **b**, FFT image of the twin-junction, the FFT images of the twins G1, G2 and G3 are shown in the bottom panel. **c**, The HAADF-STEM image of a fivefold twins. **d**, FFT image of the fivefold twins shown in (**c**). **e**, The HAADF-STEM image of a stacking fault in the Turing stripe. **f**, Strain mapping in the vicinity of the stacking fault shown in (**e**).

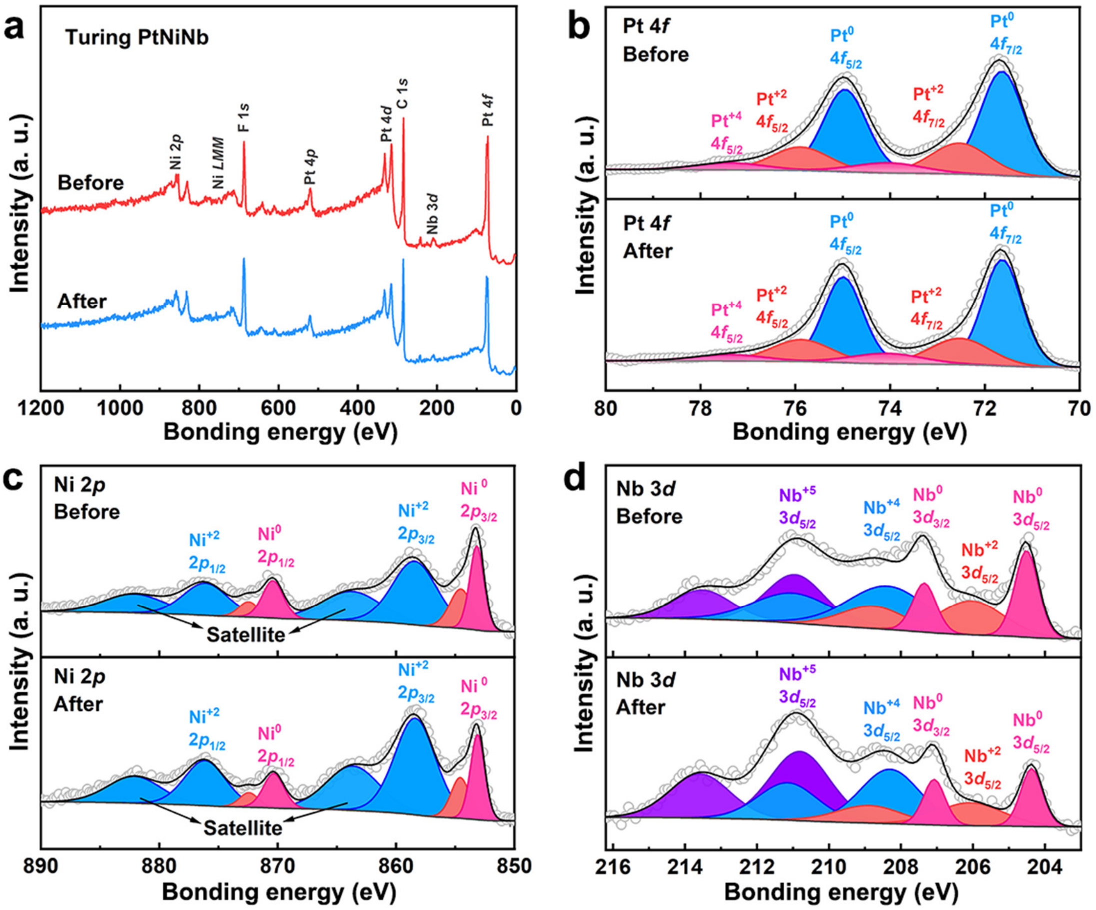

Supplementary Fig. 20 | a, XPS spectra of Turing PtNiNb before and after long-term stability test. High resolution XPS spectra comparison for **b**, Pt 4*f*, **c**, Ni 2*p* and **d**, Nb 3*d* orbitals.

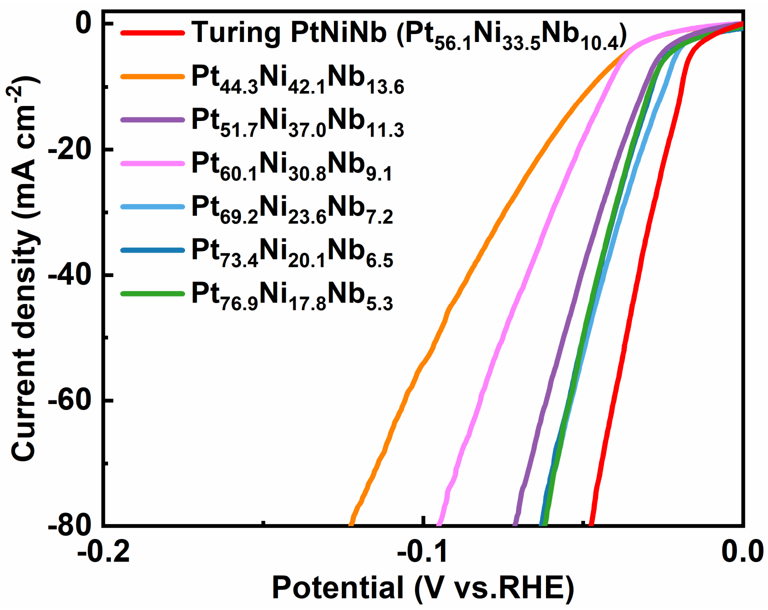

Supplementary Fig. 21 | LSV curves of the Pt-Ni-Nb nanosheets with different compositions tested in 1.0 M KOH, with 85 % resistance compensation.

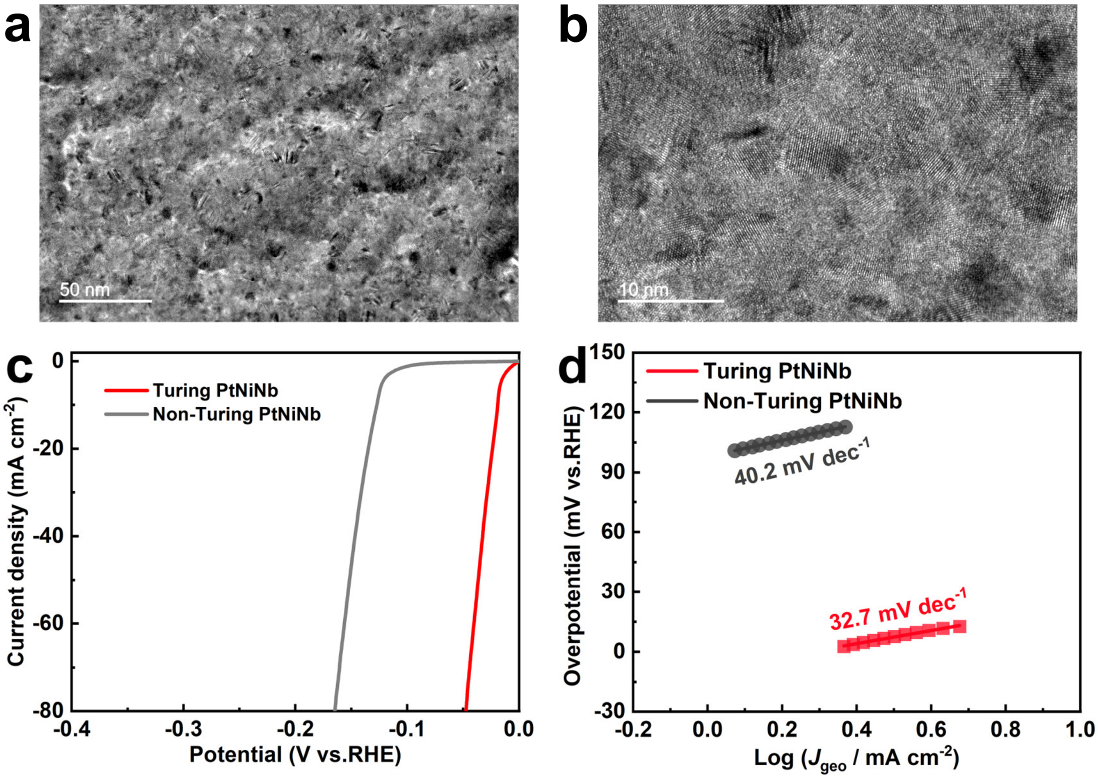

Supplementary Fig. 22 | a, TEM image of PtNiNb without Turing structure. **b**, HRTEM image of PtNiNb without Turing structure. **c**, LSV curves of Turing PtNiNb and PtNiNb without Turing structure in 1.0 M KOH. RHE, reversible hydrogen electrode. **d**, Tafel plots of Turing PtNiNb and PtNiNb without Turing structure.

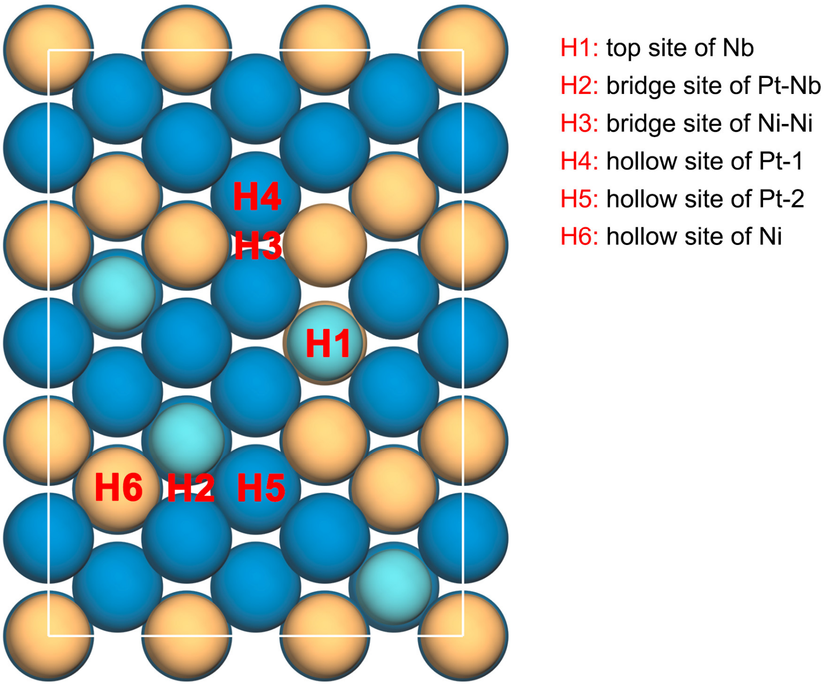

Supplementary Fig. 23 | The DFT determined active sites with ΔG_{H*} values between ± 0.200 eV on the PtNiNb slab surface.

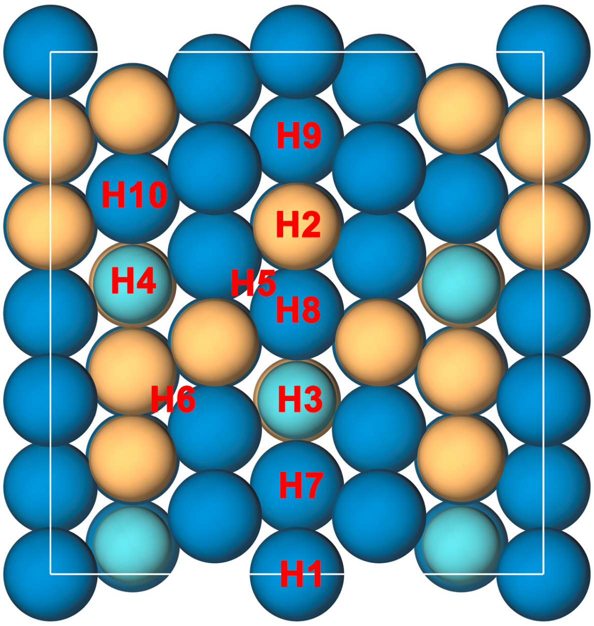

H1: top site of Pt (on twin bounadry)
H2: top site of Ni (on twin boudnary)
H3: top site of Nb-1 (on twin boudnary)
H4: top site of Nb-2 (near twin boundary)
H5: bridge site of Ni-Ni-1 (near twin boundary)
H6: bridge site of Ni-Ni-2
H7: hollow site of Pt-1 (on twin boundary)
H8: hollow site of Pt-2 (on twin boundary)
H9: hollow site of Pt-3 (on twin boundary)
H10: hollow site of Pt-4 (near twin boundary)

Supplementary Fig. 24 | The DFT determined active sites with ΔG_{H*} values between ± 0.200 eV on the PtNiNb twin slab surface.

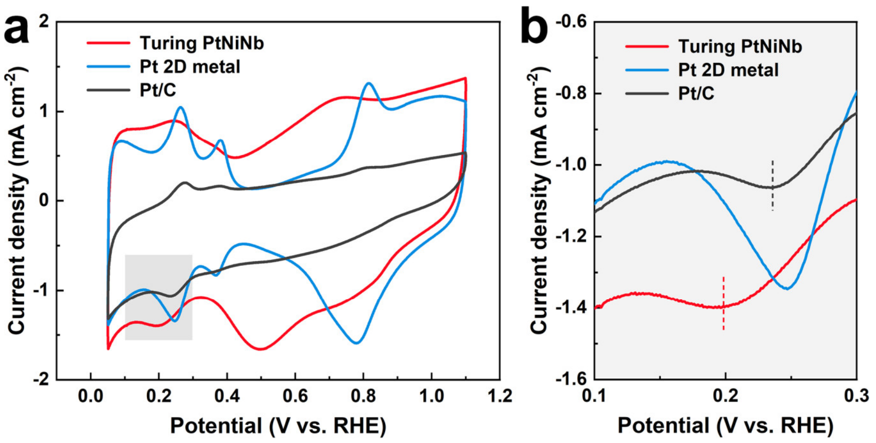

Supplementary Fig. 25 | Underpotentially deposited hydrogen (H_{upd}) peak analysis. **a**, CV curves of Turing PtNiNb, Pt 2D metal and Pt/C measured in Argon purged 1.0 M KOH at a scan rate of 50 mV s^{-1}. **b**, Enlarged CV curves of gray area in (**a**).

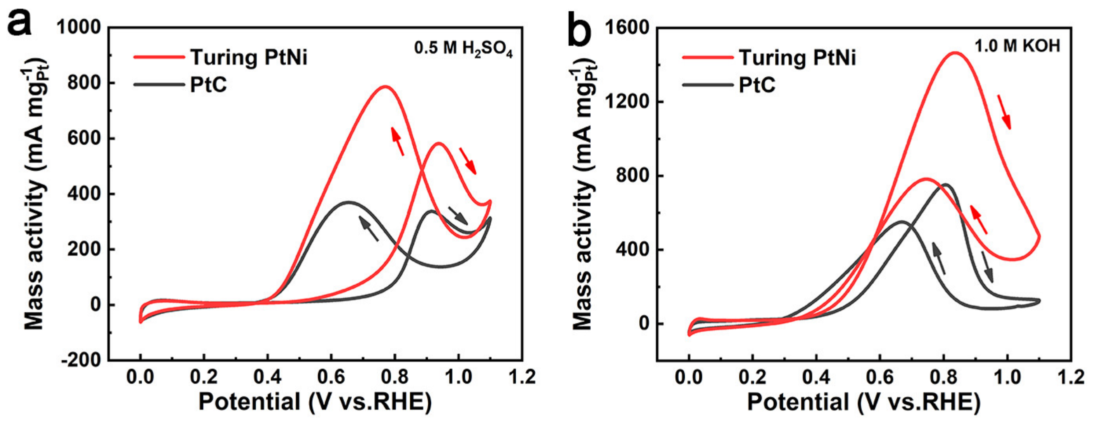

Supplementary Fig. 26 | a, CV curves of Turing PtNiNb and Pt/C at 50 mV s^{-1} in 0.5 M H_2SO_4 with 1.0 M ethanol, the inset table shows the mass activities. **b**, CV curves of Turing PtNiNb and Pt/C at 50 mV s^{-1} in 1.0 M KOH with 1.0 M ethanol.

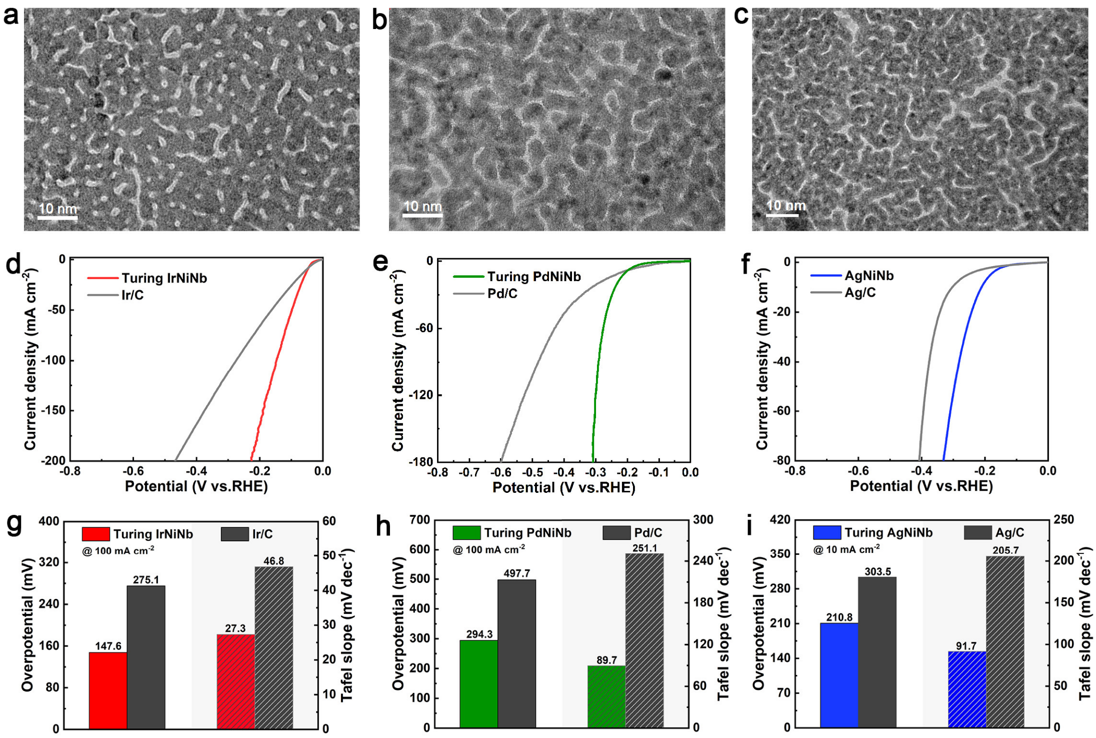

Supplementary Fig. 27 | TEM images of **a**, Turing Ir-Ni-Nb nanosheet, **b**, Turing Pd-Ni-Nb nanosheet and **c**, Turing Ag-Ni-Nb nanosheet. LSV curves measured in 1.0 M KOH of **d**, Turing Ir-Ni-Nb and 20 wt% Ir/C, **e**, Turing Pd-Ni-Nb and 20 wt% Pd/C and **f**, Turing Ag-Ni-Nb and 20 wt% Ag/C. Comparisons for overpotential and Tafel slopes between **g**, Turing Ir-Ni-Nb and 20 wt% Ir/C, **h**, Turing Pd-Ni-Nb and 20 wt% Pd/C and **i**, Turing Ag-Ni-Nb and 20 wt% Ag/C.

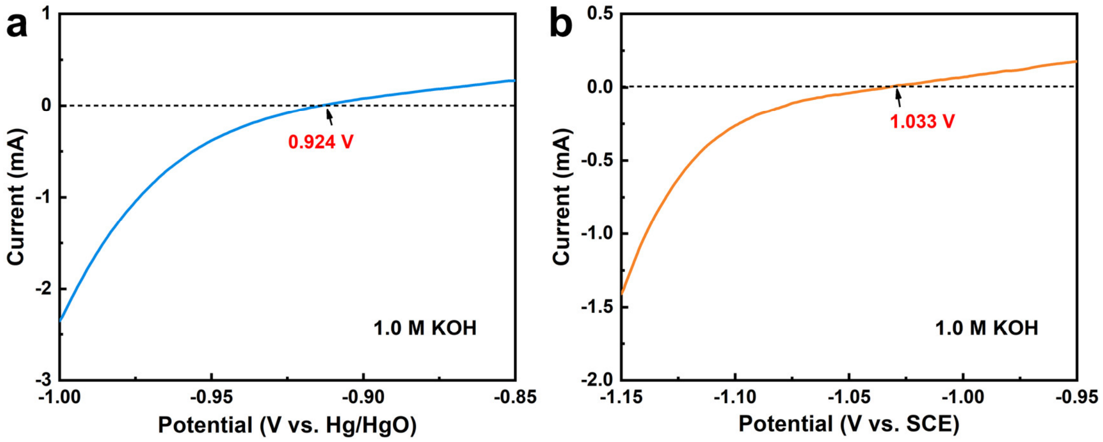

Supplementary Fig. 28 | Reference electrodes calibration based on the reported method[1,2]. LSV curves of Pt in 1.0 M KOH solution (H_2-saturated), used for calibration of **a**, Hg/HgO electrode and **b**, saturated calomel electrode with respect to RHE. Scan rate: 5 mV s^{-1}. Two Pt sheets and calibrated electrodes were used as the counter, working, and reference electrode, respectively. In 1.0 M KOH, E(RHE) = E(vs. Hg/HgO) + 0.924 V and E(RHE) = E(vs. SCE) + 1.033 V.

Supplementary Table 1. EXAFS fitting parameters at the Pt L_3-edge for various samples （S_0^2=0.84）

	shell	CN	R(Å)	σ^2	ΔE_0	R factor
Pt foil	Pt-Pt	12	2.77±0.01	0.0046	7.7±0.4	0.0023
Pt 2D metal	Pt-O	0.5±0.2	2.00±0.02	0.0044	7.8±0.6	0.0029
	Pt-Pt	9.0±0.2	2.76±0.03	0.0046		
PtNi 2D metal	Pt-Ni	4.6±0.2	2.62±0.01	0.0095	6.0±0.9	0.0030
	Pt-Pt	4.9±0.3	2.69±0.04	0.0128		
Non-Turing PtNiNb	Pt-O	0.2±0.1	1.94±0.04	0.0044	4.5±0.6	0.0074
	Pt-Ni	1.0±0.2	2.60±0.01	0.0039		
	Pt-Nb	0.4±0.1	2.65±0.01	0.0004		
	Pt-Pt	5.8±0.5	2.71±0.01	0.0102		
Turing PtNiNb	Pt-O	0.3±0.1	1.89±0.02	0.0058	4.4±0.6	0.0025
	Pt-Ni	1.0±0.3	2.59±0.01	0.0100		
	Pt-Nb	0.6±0.2	2.67±0.01	0.0071		
	Pt-Pt	6.2±0.3	2.72±0.01	0.0090		

[a]*N*: coordination numbers; [b]*R*: bond distance; [c]σ^2: Debye-Waller factors; [d] ΔE_0: the inner potential correction. *R* factor: goodness of fit.

Supplementary Table 2. EXAFS fitting parameters at the Ni K-edge for various samples （S_0^2=0.79）

	shell	CN	R(Å)	σ^2	ΔE_0	R factor
Ni foil	Ni-Ni	12	2.48±0.01	0.0061	7.3±0.4	0.0012
NiNb 2D metal	Ni-O	2.3±0.3	2.05±0.01	0.0078	1.1±2.1	0.0050
	Ni-Ni	6.8±0.4	2.52±0.02	0.0086		
	Ni-Nb	1.2±0.3	2.58±0.02	0.0036		
PtNi 2D metal	Ni-O	0.3±0.1	1.99±0.03	0.0027	-6.9±1.2	0.0068
	Ni-Ni	4.7±0.4	2.59±0.01	0.0140		
	Ni-Pt	3.1±0.3	2.62±0.01	0.0060		
Non-Turing PtNiNb	Ni-O	0.8±0.1	2.02±0.01	0.0114	-7.4±0.6	0.0051
	Ni-Ni	0.9±0.1	2.43±0.01	0.0103		
	Ni-Pt	7.2±0.4	2.64±0.01	0.0148		
	Ni-Nb	0.2±0.1	2.98±0.01	0.0010		
Turing PtNiNb	Ni-O	2.3±0.2	2.06±0.01	0.0055	-1.8±1.3	0.0091
	Ni-Pt	1.6±0.2	2.62±0.01	0.0026		
	Ni-Ni	2.8±0.2	3.06±0.02	0.0026		

[a]*N*: coordination numbers; [b]*R*: bond distance; [c]σ^2: Debye-Waller factors; [d] ΔE_0: the inner potential correction. *R* factor: goodness of fit.

Supplementary Table 3. EXAFS fitting parameters at the Nb K-edge for various samples （S_0^2=0.94）

	shell	CN	R(Å)	σ^2	ΔE_0	R factor
Nb foil	Nb-Nb	8	2.85±0.01	0.0069	4.3±0.6	0.0009
	Nb-Nb	6	3.28±0.01	0.0067		
NiNb 2D metal	Nb-O	1.4±0.1	2.06±0.02	0.0065	6.3±2.2	0.0053
	Nb-Ni	1.6±0.1	2.55±0.01	0.0057		
	Nb-Nb	4.0±0.5	3.30±0.01	0.0148		
Non-Turing PtNiNb	Nb-O	0.7±0.1	2.03±0.01	0.0053	-6.5±1.7	0.0048
	Nb-Ni	1.6±0.4	2.63±0.01	0.0095		
	Nb-Pt	5.9±1.3	2.72±0.01	0.0123		
	Nb-Nb	0.8±0.8	2.94±0.02	0.0123		
Turing PtNiNb	Nb-O	1.3±0.1	2.03±0.01	0.0020	4.3±1.9	0.0085
	Nb-Ni	0.5±0.1	2.57±0.02	0.0029		
	Nb-Pt	1.2±0.2	2.73±0.02	0.0021		
	Nb-Nb	1.6±0.2	3.35±0.02	0.0013		

[a]*N*: coordination numbers; [b]*R*: bond distance; [c]σ^2: Debye-Waller factors; [d] ΔE_0: the inner potential correction. *R* factor: goodness of fit.

Supplementary Table 4. Resistances of carbon cloth electrodes and glassy carbon electrodes.

samples	Carbon cloth electrode (ohm)	Glassy carbon electrode (ohm)
Turing PtNiNb	2.5±0.03	5.8±0.03
PtNi 2D metal	3.1±0.03	5.9±0.05
Pt 2D metal	2.9±0.05	7.6±0.03
Pt/C	2.7±0.03	11.6±0.09
NiNb 2D metal	2.9±0.07	8.4±0.05

Supplementary Table 5. Solution resistances and charge transfer resistances corresponding to samples in Fig. based on fitting results.

Samples	Solution resistance (ohm)	Charge transfer resistance (ohm)
Turing PtNiNb	6.8	5.4
PtNi 2D metal	8.9	6.8
Pt 2D metal	14.3	28.2
NiNb 2D metal	12.0	46.5

Supplementary Table 6. Electrochemical active surface area (ECSA) for Turing PtNiNb, PtNi 2D and Pt 2D metal calculated based on Cu_{upd} stripping measurement.

Samples	ECSA ($m^2\ g^{-1}_{pt}$)
Turing PtNiNb	54.1
PtNi 2D metal	20.6
Pt 2D metal	17.0

Supplementary Table 7. TOF values at certain overpotential of Turing PtNiNb, PtNi 2D metal, Pt 2D metal and other reported electrocatalysts in 1.0 M KOH.

Catalyst	Overpotential (V)	TOF (s^{-1})	Reference
Turing PtNiNb	0.1	27.53	This work
PtNi 2D metal	0.1	16.17	This work
Pt 2D metal	0.1	9.82	This work
Pt@MXene	0.1	13.08	3
Pt_1/N-C	0.05	1.89	4
Pt/np-$Co_{0.85}$Se	0.1	3.93	2
Pt/NiZrTi	0.1	0.89	5
CoNiRu-NT	0.1	0.33	6
Ru@MWCNT	0.025	0.4	7
Ir@CON	0.025	0.2	8
Ru@C_2N	0.025	0.76	9
RhPd-H NPs	0.06	0.33	10
$Ru\Delta_{c\rightarrow h}$/C	0.05	3.03	11
R-NiRu	0.1	0.78	12
RuNi/CQDs-600	0.1	5.03	13
W-$NiS_{0.5}Se_{0.5}$	0.21	0.13	14
CuCo-CAT/CC	0.221	1.5	15
$Ni_{0.8}Fe_{0.2}S_2$	0.2	0.2	16

Supplementary Table 8. Mass-normalized charge for H_2 evolution in stability measurement of Turing PtNiNb and other state-of-the-art electrocatalysts in alkaline electrolyte.

Catalyst	η_{10} (mV)	J (mA cm^{-2})	t (h)	Retention (%)	Mass loading (mg$_{PGM}$ cm^{-2})	Q_{PMG} (C g$^{-1}_{PMG}$)	Ref.
Turing PtNiNb	18	200	60	100	0.0628	53.04	This work
PtNi 2D metal	27.2	200	24	66.6	0.0648	17.14	This work
Pt 2D metal	33.6	200	24	70	0.0609	18.60	This work
Pt/C	51.0	200	24	67.3	0.065	17.15	This work
$PtTe_2$-600NSs	22	200	24	100	0.162	8.23	3
Pt_1/N-C	46	10	20	100	0.00625	8.89	4
Pt-CoS_2/CC	24	10	50	94.5	0.0365	3.70	17
Pt/np-$Co_{0.85}$Se	58	10	27.8	100	0.021	3.68	2
Pt/NiZrTi	37	10	70	100	1.024	0.19	5
Ni_3N/Pt	50	10	24	82.5	0.74	0.08	18
PtNi-O	39.8	10	10	61.6	0.0051	4.40	19
Pt_3Ni_2 NWs-S/C	42	5	5	100	0.0153	0.45	20
hcp Pt-Ni alloy[a]	65	10	1	50	0.0015	1.39	21
RuP(L-RP)	18	10	200	100	0.4	1.39	22
Ru@GnP	22	30	15	100	0.0267	4.67	23
Cu-doped Ru-RuO_2/C	28	15	11.1	100	0.285	0.16	24
RhPd-H NPs	36.6	10	4	75	0.038	0.63	10
a-$Rh(OH)_3$/NiTe	25	10	75	100	0.311	0.67	25
$Pd_4S/Pd_3P_{0.95}$	42	500	20	100	0.238	11.67	26
Ru_{NP}@RuN_x-OFC/NC	19	700	10	100	0.37	5.25	27

η_{10} refers to overpotential at current density of 10 mA cm^{-2}. *t* refers to time. *J* refers to current density. Q_{PMG} refers to mass-normalized charge. [a]The stability test was measured in 0.1 M KOH electrolyte.

Supplementary References

1. Green CL, Kucernak A. Determination of the platinum and ruthenium ssurface areas in platinum−ruthenium alloy electrocatalysts by underpotential deposition of copper. I. unsupported catalysts. *J. Phys. Chem. B* **106**, 1036-1047 (2002).

2. Jiang K, *et al.* Single platinum atoms embedded in nanoporous cobalt selenide as electrocatalyst for accelerating hydrogen evolution reaction. *Nat. Commun.* **10**, 1743 (2019).

3. Li X, *et al.* Ordered clustering of single atomic Te vacancies in atomically thin $PtTe_2$ promotes hydrogen evolution catalysis. *Nat. Commun.* **12**, 2351 (2021).

4. Fang S, *et al.* Uncovering near-free platinum single-atom dynamics during electrochemical hydrogen evolution reaction. *Nat. Commun.* **11**, 1029 (2020).

5. Li R, *et al.* Flexible honeycombed nanoporous/glassy hybrid for efficient electrocatalytic hydrogen generation. *Adv. Mater.* **31**, e1904989 (2019).

6. Wang Y, *et al.* Competitive coordination-oriented monodispersed ruthenium sites in conductive MOF/LDH hetero-nanotree catalysts for efficient overall water splitting in alkaline media. *Adv. Mater.* **34**, e2107488 (2022).

7. Kweon DH, *et al.* Ruthenium anchored on carbon nanotube electrocatalyst for hydrogen production with enhanced Faradaic efficiency. *Nat. Commun.* **11**, 1278 (2020).

8. Mahmood J, *et al.* Encapsulating iridium nanoparticles inside a 3D cage-like organic network as an efficient and durable catalyst for the hydrogen evolution reaction. *Adv. Mater.* **30**, e1805606 (2018).

9. Mahmood J, *et al.* An efficient and pH-universal ruthenium-based catalyst for the hydrogen evolution reaction. *Nat. Nanotechnol.* **12**, 441-446 (2017).

10. Fan J, *et al.* Interstitial hydrogen atom modulation to boost hydrogen evolution in Pd-based alloy nanoparticles. *ACS nano* **13**, 12987-12995 (2019).

11. Kim J, *et al.* Crystal phase ttransition creates a highly active and stable RuC_X nanosurface for hydrogen evolution reaction in alkaline media. *Adv. Mater.* **33**, e2105248 (2021).

12. Chen X, *et al.* Atomically dispersed ruthenium on nickel hydroxide ultrathin nanoribbons for highly efficient hydrogen evolution reaction in alkaline media. *Adv. Mater.* **33**, e2104764 (2021).

13. Liu Y, *et al.* A general route to prepare low-ruthenium-content bimetallic electrocatalysts for pH-universal hydrogen evolution reaction by using carbon quantum dots. *Angew. Chem. Int. Ed.* **59**, 1718-1726 (2020).

14. Wang Y*, et al.* Highly active and durable single-atom tungsten-doped $NiS_{0.5}Se_{0.5}$ nanosheet@$NiS_{0.5}Se_{0.5}$ nanorod heterostructures for water splitting. *Adv. Mater.* **34**, e2107053 (2022).

15. Geng B*, et al.* Conductive CuCo-based bimetal organic framework for efficient hydrogen evolution. *Adv. Mater.* **33**, e2106781 (2021).

16. Sun Y*, et al.* Phase reconfiguration of multivalent nickel sulfides in hydrogen evolution. *Energy Environ. Sci.* **15**, 633-644 (2022).

17. Han X*, et al.* Ultrafine Pt nanoparticle-decorated pyrite-type CoS_2 nanosheet arrays coated on carbon cloth as a bifunctional electrode for overall water splitting. *Adv. Energy Mater.* **8**, 1800935 (2018).

18. Wang Y, Chen L, Yu X, Wang Y, Zheng G. S Superb alkaline hydrogen evolution and simultaneous electricity generation by Pt-decorated Ni_3N nanosheets. *Adv. Energy Mater.* **7**, 1601390 (2017).

19. Zhao Z*, et al.* Surface-engineered PtNi-O nanostructure with record-high performance for electrocatalytic hydrogen evolution reaction. *J. Am. Chem. Soc.* **140**, 9046-9050 (2018).

20. Wang P*, et al.* Precise tuning in platinum-nickel/nickel sulfide interface nanowires for synergistic hydrogen evolution catalysis. *Nat. Commun.* **8**, 14580 (2017).

21. Cao Z*, et al.* Platinum-nickel alloy excavated nano-multipods with hexagonal close-packed structure and superior activity towards hydrogen evolution reaction. *Nat. Commun.* **8**, 15131 (2017).

22. Yu J*, et al.* Bigger is surprisingly better: agglomerates of larger RuP nanoparticles outperform benchmark Pt nanocatalysts for the hydrogen evolution reaction. *Adv. Mater.* **30**, e1800047 (2018).

23. Li F, Han GF, Noh HJ, Ahmad I, Jeon IY, Baek JB. Mechanochemically assisted synthesis of a Ru catalyst for hydrogen evolution with performance superior to Pt in both acidic and alkaline media. *Adv. Mater.* **30**, e1803676 (2018).

24. Yang K*, et al.* Ultrasmall Ru/Cu-doped RuO_2 complex embedded in amorphous carbon skeleton as highly active bifunctional electrocatalysts for overall water splitting. *Small* **14**, e1803009 (2018).

25. Sun H*, et al.* Achieving highly efficient pH-universal hydrogen evolution by superhydrophilic amorphous/crystalline $Rh(OH)_3$/NiTe coaxial nanorod array electrode. *Appl. Catal. B: Environ.* **305**, 121088 (2022).

26. Zhang G, *et al.* Interfacial engineering to construct antioxidative $Pd_4S/Pd_3P_{0.95}$ heterostructure for robust hydrogen production at high current density. *Adv. Energy Mater.* **12**, 2103511 (2022).

27. Feng Y, *et al.* Spherical vs. planar: Steering the electronic communication between Ru nanoparticle and single atom to boost the electrocatalytic hydrogen evolution activity both in acid and alkaline. *Appl. Catal. B: Environ.* **307**, 121193 (2022).
